# Effects of Sensorimotor Rhythm Modulation on the Human Flexor Carpi Radialis H-Reflex

**DOI:** 10.3389/fnins.2018.00505

**Published:** 2018-07-25

**Authors:** Aiko K. Thompson, Hannah Carruth, Rachel Haywood, N. Jeremy Hill, William A. Sarnacki, Lynn M. McCane, Jonathan R. Wolpaw, Dennis J. McFarland

**Affiliations:** ^1^Department of Health Sciences and Research, College of Health Professions, Medical University of South Carolina, Charleston, SC, United States; ^2^Division Biomedical Engineering, School of Engineering, University of Glasgow, Glasgow, United Kingdom; ^3^Burke Neurological Institute, White Plains, NY, United States; ^4^Blythedale Children's Hospital, Valhalla, NY, United States; ^5^National Center for Adaptive Neurotechnologies, Wadsworth Center, New York State Department of Health, Albany, NY, United States; ^6^Albany Stratton VA Medical Center, Albany, NY, United States

**Keywords:** EEG mu-rhythm, H-reflex, brain-computer interface (BCI), spinal cord injuries, task-dependent adaptation

## Abstract

People can learn over training sessions to increase or decrease sensorimotor rhythms (SMRs) in the electroencephalogram (EEG). Activity-dependent brain plasticity is thought to guide spinal plasticity during motor skill learning; thus, SMR training may affect spinal reflexes and thereby influence motor control. To test this hypothesis, we investigated the effects of learned mu (8–13 Hz) SMR modulation on the flexor carpi radialis (FCR) H-reflex in 6 subjects with no known neurological conditions and 2 subjects with chronic incomplete spinal cord injury (SCI). All subjects had learned and practiced over more than 10 < 30-min training sessions to increase (SMR-up trials) and decrease (SMR-down trials) mu-rhythm amplitude over the hand/arm area of left sensorimotor cortex with ≥80% accuracy. Right FCR H-reflexes were elicited at random times during SMR-up and SMR-down trials, and in between trials. SMR modulation affected H-reflex size. In all the neurologically normal subjects, the H-reflex was significantly larger [116% ± 6 (mean ± SE)] during SMR-up trials than between trials, and significantly smaller (92% ± 1) during SMR-down trials than between trials (*p* < 0.05 for both, paired *t*-test). One subject with SCI showed similar H-reflex size dependence (high for SMR-up trials, low for SMR-down trials): the other subject with SCI showed no dependence. These results support the hypothesis that SMR modulation has predictable effects on spinal reflex excitability in people who are neurologically normal; they also suggest that it might be used to enhance therapies that seek to improve functional recovery in some individuals with SCI or other CNS disorders.

## Introduction

The past several decades of non-invasive brain-computer interface (BCI) research show that people can learn through a series of brief training sessions to control mu (8–13 Hz) and/or beta (18–26 Hz) sensorimotor rhythms (SMR) recorded by electroencephalogram (EEG) over sensorimotor cortex (Wolpaw et al., 1991; Wolpaw and McFarland, 1994). Such BCI-based SMR training might help to improve motor function recovery in people with CNS disorders by guiding activity-dependent brain plasticity (Dobkin, 2007; Daly and Wolpaw, 2008). Boulay et al. (2011) showed that trained SMR control affects reaction time, indicating that SMR modulation influences a simple motor performance. Furthermore, activity-dependent brain plasticity is thought to guide the spinal cord plasticity that contributes to motor skill learning (Wolpaw, 2010). To determine whether SMR modulation might be used to guide spinal cord plasticity so as to enhance functional recovery, the present study explored the impact of SMR amplitude on the size of the H-reflex (an electrical analog of spinal stretch reflex) in the forearm muscle flexor carpi radialis (FCR).

## Materials and methods

### Study overview

The subjects were 6 people with no known neurological conditions (5 men and 1 woman; age 22–68 years) and two people with stable chronic incomplete spinal cord injury (SCI) [a 42-year-old man with a 2-yr-old incomplete SCI (AIS: American Spinal Injury Association Impairment Scale D) at C4 and a 33-year-old woman with a 10-yr-old incomplete SCI (AIS D) at C5-7]. Both people with SCI had been on stable doses of baclofen for >6 months prior to their study participation. Their inclusion was intended to provide some initial insight into the therapeutic potential of SMR training. The study was reviewed and approved by the Institutional Review Boards of Helen Hayes Hospital and the Wadsworth Center, New York State Department of Health. All subjects provided informed consent.

**Figure 1:**
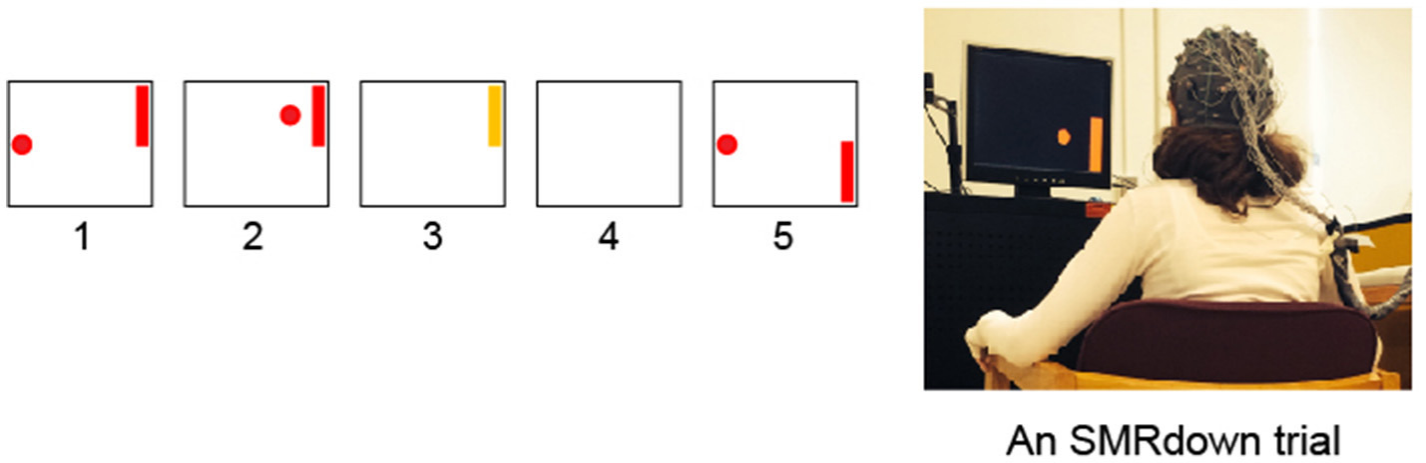
Subjects learn over a series of training sessions to use SMR amplitudes in the μ (8–12 Hz) frequency band over left sensorimotor cortex (at/around the C3 or CP3 electrodes) to move a cursor vertically while it moves from left to right at a constant rate (Wolpaw et al., 1991; Wolpaw and McFarland, 1994; McFarland et al., 2003). (1) a target appears; (2) 1 s later the cursor appears and moves in two dimensions with vertical movement controlled by the subject's SMR amplitude; (3) the cursor reaches the target and the target flashes for 1 s; (4) the screen is blank for 1 s; and then (5) the next trial begins. (If the cursor misses the target, the flash does not occur, the screen simply goes blank for 2 s.) In each training session, the participant goes through 10 blocks of ≈18 SMR trials each, separated by ≥1-min rest periods.

First, each subject learned and practiced over >10 training sessions (< 30 min/session, 2–3 sessions/week) of a BCI cursor-control task (Figure 1, fully described in, Wolpaw and McFarland, 1994, 2004; McFarland et al., 2003) to increase (SMR-up trials) and decrease (SMR-down trials) mu-rhythm (8–13 Hz) amplitude over the hand/arm area of left sensorimotor cortex (electrode C3 or CP3, Jasper, 1958; Ebner et al., 1999; Nuwer et al., 1999; Jurcak et al., 2007) with ≥80% accuracy. The number of sessions before reaching ≥80% cursor control accuracy varied across subjects (from 2 to 8). Regardless of how soon the ≥80% accuracy was achieved, all subjects completed at least 10 training sessions. In these sessions, 32 channels of EEG were collected with active electrodes (g.tec Medical Engineering GMBH, Austria) and the general-purpose BCI software platform BCI2000 (Schalk et al., 2004). EEG was sampled at 256 Hz, referenced to the left earlobe (ground at the forehead electrode AFz) (Jasper, 1958; Ebner et al., 1999; Nuwer et al., 1999; Jurcak et al., 2007). EEG features (logarithms of the amplitudes in 3-Hz-wide frequency bands) were extracted by a surface-Laplacian spatial filter (McFarland et al., 1997) and autoregressive spectral estimation (model order 16) (Marple, 1987; McFarland and Wolpaw, 2008). Features in the mu-rhythm frequency range at C3 or CP3 controlled cursor movement. Every 100-ms, their values for the previous 200-ms segment were calculated and converted into vertical cursor movement by a linear equation. At the beginning of each cursor-movement trial, a target appeared randomly at the top right or the bottom right of the screen and the cursor appeared in the middle of the left edge of the screen. The cursor moved from left to right at a constant rate; the subject learned to control SMR amplitude to move the cursor up or down so that it hit the target when it reached the right edge. Each training session included 10 blocks of 16-18 trials each.

After >10 training sessions, (i.e., fully trained to hit the target at ≥80% accuracy), the subject began the H-reflex component of the study in which the right FCR H-reflex was elicited during SMRup and SMRdown trials and in between trials.

### H-reflex recording

Surface EMG activity from FCR and its antagonist extensor carpi radialis (ECR) was amplified, band-pass filtered (10–1,000 Hz), and sampled at 3,200 Hz. To elicit the FCR H-reflex, the median nerve was stimulated in the cubital fossa, using surface Ag-AgCl electrodes (2.2 × 2.2 cm) and 0.5-ms square pulses. Stimulation was delivered when the subject had maintained 5–15% maximum voluntary contraction (MVC) level of FCR EMG activity with resting level ECR activity (typically < 10 μV) for at least 2 s. For all H-reflex measurements, the subject's right arm was strapped to a custom-made arm support platform with the shoulder at ≈90° in the sagittal plane and ≈40° in the transverse plane, the elbow at full extension, and the hand in full supination.

To determine the stimulus intensity that elicited a submaximal H-reflex with a small M-wave, the FCR H-reflex/M-wave recruitment curve was obtained while the subject maintained the preset levels of FCR and ECR EMG activity (Zehr and Stein, 1999; Kido et al., 2004). This stimulus intensity was used to elicit the H-reflex during SMRup trials, SMRdown trials, and in between trials (see below). Then, the subject with right arm on the platform completed; a block of 16–18 SMRup or SMRdown trials with no voluntary EMG activation (i.e., similar to the SMR cursor task training sessions except for the arm and hand position); and a second block of 16–18 SMRup or SMRdown trials with the preset levels of FCR and ECR EMG activity without H-reflex elicitation. After these blocks confirmed that the subject was able to perform the SMR cursor task with ≥80% accuracy in this arm-hand position while maintaining the preset levels of FCR and ECR EMG activity, FCR H-reflex testing began.

While the subject performed cursor-movement trials [i.e., trials that required SMR increase (SMRup) or decrease (SMRdown)], median nerve stimulation occurred at random times during the trials and in between trials when FCR and ECR EMG activity met the preset requirements. About 30 H-reflexes were obtained from each subject in each of the three conditions (SMRup trials, SMRdown trials, in between trials).

### Data analysis

Rectified EMG activity in the 50-ms pre-stimulus period was averaged for each trial to measure the background activity level. The FCR H-reflex and the M-wave amplitudes were measured as peak-to-peak values in time windows determined for each subject. Typical time windows were 3-13 ms post-stimulus for the M-wave and 18–27 ms for the H-reflex. To ensure that the H-reflexes were measured with the same background EMG levels and the same stimulus intensity in all three SMR conditions, FCR and ECR background EMG and M-wave size were compared across the three conditions for each subject. Any trials that occurred with too large or small M-waves or background EMG levels were eliminated from the analysis. After removing these trials, 15–30 trials were averaged for each condition of each subject.

## Results

Background EMG levels and M-wave size did not differ among SMRup, SMRdown, and in between conditions (*p* > 0.12 for FCR and ECR background EMG and the M-wave size by repeated measures ANOVA). Thus, the difference in H-reflex size among conditions can be confidently attributed to the SMR control.

**Figure 2:**
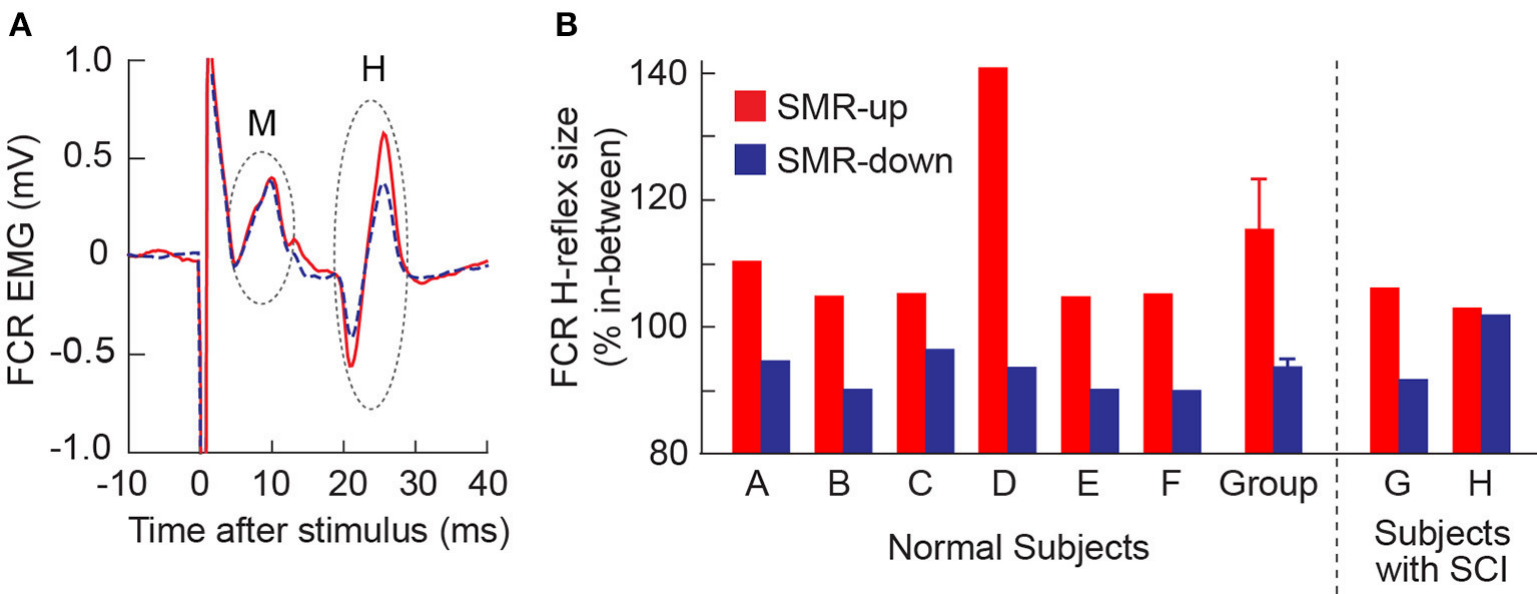
Effects of learned SMR control on the FCR H-reflex. **(A)** FCR H-reflex during the SMRup (red, solid) and SMRdown (blue, dashed) trials in subject D. About 20 responses were averaged together for each sweep. **(B)** Average FCR H-reflex sizes during SMRup (red) and SMRdown (blue) trials in normal subjects (A–F) and subjects with SCI (G and H). Group data for normal subjects are also included: H-reflex size averages 116 ± 6 (mean ± SE)% for SMRup trials and 92 ± 1% for SMRdown trials.

In all 6 normal subjects, the FCR H-reflex was larger during SMRup trials and smaller during SMRdown trials, compared with in between trials. Figure 2A shows typical H-reflex responses during SMRup and SMRdown trials. A repeated-measures ANOVA and *t*-test with Bonferroni correction showed that H-reflex sizes in SMRup and SMRdown trials differed significantly from each other (*p* = 0.02 by ANOVA and *p* = 0.0068 by *t*-test). Figure 2B displays SMRup and SMRdown H-reflex sizes normalized to the between-trial H-reflex size in individual subjects. Group mean ± SE H-reflex size was 116 ± 6(SE)% for SMRup and 92 ± 1% for SMRdown trials.

In one of the subjects with SCI (Subject G), FCR H-reflex was modulated across the three SMR conditions (Figure 2B) as it was in normal subjects. In the other person with SCI (Subject H), H-reflex modulation across the conditions was not significant.

## Discussion

SMR activity in the μ and β rhythm frequency range decreases before and during active movement (Pfurtscheller, 1989; Pfurtscheller and Neuper, 1994; Pfurtscheller et al., 2006; Klimesch et al., 2007; Boulay et al., 2011; McFarland et al., 2015). Such SMR decrease, called event-related desynchronization (ERD), is also associated with motor imagery (McFarland et al., 2000). Indeed, in the initial stages of BCI-based SMR training, people often imagine moving (or not moving) to decrease (or increase) SMR amplitude (Wolpaw and McFarland, 2004). As they acquire SMR control, such imagery tends to drop away (Wolpaw and McFarland, 2004). Motor imagery increases corticospinal excitability (Kasai et al., 1997; Rossini et al., 1999; Stinear and Byblow, 2004; Stinear et al., 2006a,b; Bakker et al., 2008; Kang et al., 2011; Gündüz and Kiziltan, 2015; Kato et al., 2015; Im et al., 2016; Tatemoto et al., 2017) and resting motoneuron excitability (Gündüz and Kiziltan, 2015). Studies of the impact of motor imagery on the H-reflex are less consistent in their results (Oishi et al., 1994; Abbruzzese et al., 1996; Yahagi et al., 1996; Bonnet et al., 1997; Kasai et al., 1997; Hashimoto and Rothwell, 1999; Hale et al., 2003; Patuzzo et al., 2003; Cowley et al., 2008; Aoyama and Kaneko, 2011; Jarjees and Vuckovic, 2016). The discrepancies among studies probably reflect differences in the imagery (e.g., visual vs. kinesthetic, Neuper et al., 2005), as well as in H-reflex testing methods. Many studies measure the H-reflex when the muscle is inactive; they do not control for subthreshold changes in motoneuron excitability, which may markedly affect H-reflex size (Stein and Thompson, 2006).

In the present study, the FCR H-reflex was always measured in the presence of a given level of ongoing FCR EMG activity and M-wave size was kept stable; thus, motoneuron pool excitability and effective stimulus intensity were the same across the three SMR conditions (i.e., SMRup trials, SMRdown trials, and in between trials). The results were quite clear: the H-reflex was larger when SMR amplitude was high and smaller when SMR amplitude was low. Boulay et al. (2015) found a similar positive correlation between H-reflex size and SMR amplitude in rats.

In general, SMR amplitude in the mu-beta range is inversely correlated with cortical activation; high SMR [i.e., event-related synchronization (ERS)] reflects cortical inhibition, low SMR (ERD) reflects cortical activation (reviewed in Klimesch et al., 2007). SMR in the hand area of sensorimotor cortices decreases during movement planning or execution (Pfurtscheller, 1989; Pfurtscheller and Neuper, 1994; Pfurtscheller et al., 2006); and voluntary modulation of pre-movement SMR affects subsequent behavior (Boulay et al., 2011; McFarland et al., 2015). When SMR amplitude decreases, cortical drive to spinal motoneurons increases (Rossini et al., 1991; Rau et al., 2003; Zarkowski et al., 2006; Sauseng et al., 2009; Takemi et al., 2013). When cortical drive to the motoneurons is increased by demanding motor tasks [e.g., beam-walking vs. treadmill-walking, (Llewellyn et al., 1990), greater postural complexity during standing (Yamashita and Moritani, 1989)], the H-reflex is smaller. This H-reflex suppression is thought to be mediated through corticospinal excitation of Ia inhibitory interneurons (Iles and Pisini, 1992; Nielsen et al., 1993) and/or interneurons affecting presynaptic inhibition of Ia afferents (Iles, 1996; Meunier and Pierrot-Deseilligny, 1998; see also Chen and Wolpaw, 2002; Chen et al., 2002).

Because learned SMR control can influence H-reflex size, it might serve as an aid in operant conditioning of the H-reflex, which can help to improve impaired locomotion after incomplete SCI (Manella et al., 2013; Thompson et al., 2013). As discussed by Thompson et al. (2009), an operant conditioning protocol can increase or decrease a targeted H-reflex. H-reflex change has two components: task-dependent adaptation that begins in several sessions and is thought to reflect plasticity in the brain; and subsequent long-term change that progresses gradually over sessions, is thought to reflect spinal plasticity, and persists after conditioning ends (Thompson et al., 2009). In this context, it is interesting to note that the magnitude of H-reflex change found in the present study (i.e., Figure 2B), is similar to the magnitude of task-dependent adaptation in the H-reflex produced by the H-reflex operant conditioning protocol (Thompson et al., 2009). This suggests that SMR training might be used to guide, and possibly even enhance, task-dependent adaptation in the H-reflex. It might thereby increase the conditioning success rate and augment the rapidity and magnitude of the long-term spinal plasticity that can trigger wider plasticity so as to improve complex motor functions (e.g., locomotion) after SCI or in other disorders (Thompson et al., 2013; Wolpaw, 2018). Such effects could increase the clinical efficacy and practicality of spinal reflex operant conditioning protocols.

## Author contributions

AT, NH, and DM designed the study. AT, HC, RH, WS, LM, and DM collected the data. AT, HC, JW, and DM conducted the analysis. AT, HC, NH, JW, and DM interpreted the results and wrote the manuscript. HC and WS performed the majority of subject training sessions prior to the main experiments conducted in this study. All authors reviewed the manuscript and approved the final version of the manuscript.

### Conflict of interest statement

The authors declare that the research was conducted in the absence of any commercial or financial relationships that could be construed as a potential conflict of interest.

## References

[B1] Abbruzzese G. Trompetto C. Schieppati M. (1996). The excitability of the human motor cortex increases during execution and mental imagination of sequential but not repetitive finger movements. Exp. Brain Res. 111, 465–472. 10.1007/BF00228736 8911941

[B2] Aoyama T. Kaneko F. (2011). The effect of motor imagery on gain modulation of the spinal reflex. Brain Res. 1372, 41–48. 10.1016/j.brainres.2010.11.023 21094636

[B3] Bakker M. Overeem S. Snijders A. H. Borm G. van Elswijk G. Toni I.. (2008). Motor imagery of foot dorsiflexion and gait: effects on corticospinal excitability. Clin. Neurophysiol. 119, 2519–2527. 10.1016/j.clinph.2008.07.282 18838294

[B4] Bonnet M. Decety J. Jeannerod M. Requin J. (1997). Mental simulation of an action modulates the excitability of spinal reflex pathways in man. Brain Res. Cogn. Brain Res. 5, 221–228. 10.1016/S0926-6410(96)00072-9 9088558

[B5] Boulay C. B. Chen X. Y. Wolpaw J. R. (2015). Electrocorticographic activity over sensorimotor cortex and motor function in awake behaving rats. J. Neurophysiol. 113, 2232–2241. 10.1152/jn.00677.2014 25632076 PMC4416631

[B6] Boulay C. B. Sarnacki W. A. Wolpaw J. R. McFarland D. J. (2011). Trained modulation of sensorimotor rhythms can affect reaction time. Clin. Neurophysiol. 122, 1820–1826. 10.1016/j.clinph.2011.02.016 21411366 PMC3132832

[B7] Chen X. Y. Wolpaw J. R. (2002). Probable corticospinal tract control of spinal cord plasticity in the rat. J. Neurophysiol. 87, 645–652. 10.1152/jn.00391.2001 11826033

[B8] Chen X. Y. Chen L. Wolpaw J. R. Jakeman L. B. (2002). Corticospinal tract transection reduces H-reflex circadian rhythm in rats. Brain Res. 942, 101–108. 10.1016/S0006-8993(02)02702-6 12031858

[B9] Cowley P. M. Clark B. C. Ploutz-Snyder L. L. (2008). Kinesthetic motor imagery and spinal excitability: the effect of contraction intensity and spatial localization. Clin. Neurophysiol. 119, 1849–1856. 10.1016/j.clinph.2008.04.004 18486544

[B10] Daly J. J. Wolpaw J. R. (2008). Brain-computer interfaces in neurological rehabilitation. Lancet Neurol. 7, 1032–1043. 10.1016/S1474-4422(08)70223-0 18835541

[B11] Dobkin B. H. (2007). Brain-computer interface technology as a tool to augment plasticity and outcomes for neurological rehabilitation. J. Physiol. 579, 637–642. 10.1113/jphysiol.2006.123067 17095557 PMC2151380

[B12] Ebner A. Sciarretta G. Epstein C. M. Nuwer M. (1999). EEG instrumentation. The International Federation of Clinical Neurophysiology. Electroencephal. Clin. Neurophysiol. Suppl 52, 7–10. 10590971

[B13] Gündüz A. Kiziltan M. E. (2015). F-wave and motor-evoked potentials during motor imagery and observation in apraxia of Parkinson disease. Muscle Nerve 52, 1072–1077. 10.1002/mus.24663 25809124

[B14] Hale B. S. Raglin J. S. Koceja D. M. (2003). Effect of mental imagery of a motor task on the Hoffmann reflex. Behav. Brain Res. 142, 81–87. 10.1016/S0166-4328(02)00397-2 12798268

[B15] Hashimoto R. Rothwell J. C. (1999). Dynamic changes in corticospinal excitability during motor imagery. Exp. Brain Res. 125, 75–81. 10.1007/s002210050660 10100979

[B16] Iles J. F. (1996). Evidence for cutaneous and corticospinal modulation of presynaptic inhibition of Ia afferents from the human lower limb. J. Physiol. 491(Pt 1), 197–207. 10.1113/jphysiol.1996.sp021207 9011611 PMC1158770

[B17] Iles J. F. Pisini J. V. (1992). Cortical modulation of transmission in spinal reflex pathways of man. J. Physiol. 455, 425–446. 10.1113/jphysiol.1992.sp019309 1336554 PMC1175652

[B18] Im H. Ku J. Kim H. J. Kang Y. J. (2016). Virtual reality-guided motor imagery increases corticomotor excitability in healthy volunteers and stroke patients. Ann. Rehabil. Med. 40, 420–431. 10.5535/arm.2016.40.3.420 27446778 PMC4951360

[B19] Jarjees M. Vucković A. (2016). The effect of voluntary modulation of the sensory-motor rhythm during different mental tasks on H reflex. Int. J. Psychophysiol. 106, 65–76. 10.1016/j.ijpsycho.2016.06.005 27318009

[B20] Jasper H. H. (1958). Report of the committee on methods of clinical examination in electroencephalography: 1957. Electroencephalogr. Clin. Neurophysiol. 10, 370–375. 10.1016/0013-4694(58)90053-1

[B21] Jurcak V. Tsuzuki D. Dan I. (2007). 10/20, 10/10, and 10/5 systems revisited: their validity as relative head-surface-based positioning systems. Neuroimage 34, 1600–1611. 10.1016/j.neuroimage.2006.09.024 17207640

[B22] Kang Y. J. Ku J. Kim H. J. Park H. K. (2011). Facilitation of corticospinal excitability according to motor imagery and mirror therapy in healthy subjects and stroke patients. Ann. Rehabil. Med. 35, 747–758. 10.5535/arm.2011.35.6.747 22506202 PMC3309378

[B23] Kasai T. Kawai S. Kawanishi M. Yahagi S. (1997). Evidence for facilitation of motor evoked potentials (MEPs) induced by motor imagery. Brain Res 744, 147–150. 10.1016/S0006-8993(96)01101-8 9030424

[B24] Kato K. Watanabe J. Muraoka T. Kanosue K. (2015). Motor imagery of voluntary muscle relaxation induces temporal reduction of corticospinal excitability. Neurosci. Res. 92, 39–45. 10.1016/j.neures.2014.10.013 25448688

[B25] Kido A. Tanaka N. Stein R. B. (2004). Spinal excitation and inhibition decrease as humans age. Can. J. Physiol. Pharmacol. 82, 238–248. 10.1139/y04-017 15181462

[B26] Klimesch W. Sauseng P. Hanslmayr S. (2007). EEG alpha oscillations: the inhibition-timing hypothesis. Brain Res. Rev. 53, 63–88. 10.1016/j.brainresrev.2006.06.003 16887192

[B27] Llewellyn M. Yang J. F. Prochazka A. (1990). Human H-reflexes are smaller in difficult beam walking than in normal treadmill walking. Exp. Brain Res. 83, 22–28. 10.1007/BF00232189 2073943

[B28] Manella K. J. Roach K. E. Field-Fote E. C. (2013). Operant conditioning to increase ankle control or decrease reflex excitability improves reflex modulation and walking function in chronic spinal cord injury. J. Neurophysiol. 109, 2666–2679. 10.1152/jn.01039.2011 23468393

[B29] Marple S. (1987). Digital Spectral Analysis with Applications. Englewood Cliffs, NJ: Prentice-Hall.

[B30] McFarland D. J. Wolpaw J. R. (2008). Sensorimotor rhythm-based brain-computer interface (BCI): model order selection for autoregressive spectral analysis. J. Neural Eng. 5, 155–162. 10.1088/1741-2560/5/2/006 18430974 PMC2747265

[B31] McFarland D. J. McCane L. M. David S. V. Wolpaw J. R. (1997). Spatial filter selection for EEG-based communication. Electroencephalogr. Clin. Neurophysiol. 103, 386–394. 10.1016/S0013-4694(97)00022-2 9305287

[B32] McFarland D. J. Miner L. A. Vaughan T. M. Wolpaw J. R. (2000). Mu and beta rhythm topographies during motor imagery and actual movements. Brain Topogr. 12, 177–186. 10.1023/A:1023437823106 10791681

[B33] McFarland D. J. Sarnacki W. A. Wolpaw J. R. (2003). Brain-computer interface (BCI) operation: optimizing information transfer rates. Biol. Psychol. 63, 237–251. 10.1016/S0301-0511(03)00073-5 12853169

[B34] McFarland D. J. Sarnacki W. A. Wolpaw J. R. (2015). Effects of training pre-movement sensorimotor rhythms on behavioral performance. J. Neural Eng. 12:066021. 10.1088/1741-2560/12/6/066021 26529119 PMC4843806

[B35] Meunier S. Pierrot-Deseilligny E. (1998). Cortical control of presynaptic inhibition of Ia afferents in humans. Exper. Brain Res. 119, 415–426. 10.1007/s002210050357 9588776

[B36] Neuper C. Scherer R. Reiner M. Pfurtscheller G. (2005). Imagery of motor actions: differential effects of kinesthetic and visual-motor mode of imagery in single-trial EEG. Brain Res. Cogn. Brain Res. 25, 668–677. 10.1016/j.cogbrainres.2005.08.014 16236487

[B37] Nielsen J. Petersen N. Deuschl G. Ballegaard M. (1993). Task-related changes in the effect of magnetic brain stimulation on spinal neurones in man. J. Physiol. 471, 223–243. 10.1113/jphysiol.1993.sp019899 8120805 PMC1143960

[B38] Nuwer M. R. Comi G. Emerson R. Fuglsang-Frederiksen A. Guérit J. M. Hinrichs H.. (1999). IFCN standards for digital recording of clinical EEG. The International Federation of Clinical Neurophysiology. Electroencephalogr. Clin. Neurophysiol. Suppl. 52, 11–14. 10590972

[B39] Oishi K. Kimura M. Yasukawa M. Yoneda T. Maeshima T. (1994). Amplitude reduction of H-reflex during mental movement simulation in elite athletes. Behav. Brain. Res. 62, 55–61. 10.1016/0166-4328(94)90037-X 7917033

[B40] Patuzzo S. Fiaschi A. Manganotti P. (2003). Modulation of motor cortex excitability in the left hemisphere during action observation: a single- and paired-pulse transcranial magnetic stimulation study of self- and non-self-action observation. Neuropsychologia 41, 1272–1278. 10.1016/S0028-3932(02)00293-2 12753966

[B41] Pfurtscheller G. (1989). Functional topography during sensorimotor activation studied with event-related desynchronization mapping. J. Clin. Neurophysiol. 6, 75–84. 10.1097/00004691-198901000-00003 2915031

[B42] Pfurtscheller G. Neuper C. (1994). Event-related synchronization of mu rhythm in the EEG over the cortical hand area in man. Neurosci. Lett. 174, 93–96. 10.1016/0304-3940(94)90127-9 7970165

[B43] Pfurtscheller G. Brunner C. Schlögl A. Lopes da Silva F. H. (2006). Mu rhythm (de)synchronization and EEG single-trial classification of different motor imagery tasks. Neuroimage 31, 153–159. 10.1016/j.neuroimage.2005.12.003 16443377

[B44] Rau C. Plewnia C. Hummel F. Gerloff C. (2003). Event-related desynchronization and excitability of the ipsilateral motor cortex during simple self-paced finger movements. Clin. Neurophysiol. 114, 1819–1826. 10.1016/S1388-2457(03)00174-3 14499743

[B45] Rossini P. M. Desiato M. T. Lavaroni F. Caramia M. D. (1991). Brain excitability and electroencephalographic activation: non-invasive evaluation in healthy humans via transcranial magnetic stimulation. Brain Res. 567, 111–119. 10.1016/0006-8993(91)91442-4 1815819

[B46] Rossini P. M. Rossi S. Pasqualetti P. Tecchio F. (1999). Corticospinal excitability modulation to hand muscles during movement imagery. Cereb. Cortex 9, 161–167. 10.1093/cercor/9.2.161 10220228

[B47] Sauseng P. Klimesch W. Gerloff C. Hummel F. C. (2009). Spontaneous locally restricted EEG alpha activity determines cortical excitability in the motor cortex. Neuropsychologia 47, 284–288. 10.1016/j.neuropsychologia.2008.07.021 18722393

[B48] Schalk G. McFarland D. J. Hinterberger T. Birbaumer N. Wolpaw J. R. (2004). BCI2000: a general-purpose brain-computer interface (BCI) system. IEEE Trans. Biomed. Eng. 51, 1034–1043. 10.1109/TBME.2004.827072 15188875

[B49] Stein R. B. Thompson A. K. (2006). Muscle reflexes in motion: how, what, and why? Exerc. Sport Sci. Rev. 34, 145–153. 10.1249/01.jes.0000240024.37996.e5 17031251

[B50] Stinear C. M. Byblow W. D. (2004). Modulation of corticospinal excitability and intracortical inhibition during motor imagery is task-dependent. Exp. Brain Res. 157, 351–358. 10.1007/s00221-004-1851-z 14997259

[B51] Stinear C. M. Byblow W. D. Steyvers M. Levin O. Swinnen S. P. (2006a). Kinesthetic, but not visual, motor imagery modulates corticomotor excitability. Exp. Brain Res. 168, 157–164. 10.1007/s00221-005-0078-y 16078024

[B52] Stinear C. M. Fleming M. K. Byblow W. D. (2006b). Lateralization of unimanual and bimanual motor imagery. Brain Res. 1095, 139–147. 10.1016/j.brainres.2006.04.008 16713588

[B53] Takemi M. Masakado Y. Liu M. Ushiba J. (2013). Event-related desynchronization reflects downregulation of intracortical inhibition in human primary motor cortex. J. Neurophysiol. 110, 1158–1166. 10.1152/jn.01092.2012 23761697

[B54] Tatemoto T. Tsuchiya J. Numata A. Osawa R. Yamaguchi T. Tanabe S.. (2017). Real-time changes in corticospinal excitability related to motor imagery of a force control task. Behav. Brain Res. 335, 185–190. 10.1016/j.bbr.2017.08.020 28827129

[B55] Thompson A. K. Chen X. Y. Wolpaw J. R. (2009). Acquisition of a simple motor skill: task-dependent adaptation plus long-term change in the human soleus H-reflex. J. Neurosci. 29, 5784–5792. 10.1523/JNEUROSCI.4326-08.2009 19420246 PMC2696311

[B56] Thompson A. K. Pomerantz F. R. Wolpaw J. R. (2013). Operant conditioning of a spinal reflex can improve locomotion after spinal cord injury in humans. J. Neurosci. 33, 2365–2375. 10.1523/JNEUROSCI.3968-12.2013 23392666 PMC3579496

[B57] Wolpaw J. R. (2010). What can the spinal cord teach us about learning and memory? Neuroscientist 16, 532–549. 10.1177/1073858410368314 20889964

[B58] Wolpaw J. R. (2018). The negotiated equilibrium model of spinal cord functiontiated equilibrium model of spinal cord function. J. Physiol. 10.1113/JP275532. [Epub ahead of print] 29663410 PMC6092289

[B59] Wolpaw J. R. McFarland D. J. (1994). Multichannel EEG-based brain-computer communication. Electroencephalogr. Clin. Neurophysiol. 90, 444–449. 10.1016/0013-4694(94)90135-X 7515787

[B60] Wolpaw J. R. McFarland D. J. (2004). Control of a two-dimensional movement signal by a noninvasive brain-computer interface in humans. Proc. Natl. Acad. Sci. U.S.A. 101, 17849–17854. 10.1073/pnas.0403504101 15585584 PMC535103

[B61] Wolpaw J. R. McFarland D. J. Neat G. W. Forneris C. A. (1991). An EEG-based brain-computer interface for cursor control. Electroencephalogr. Clin. Neurophysiol 78, 252–259. 10.1016/0013-4694(91)90040-B 1707798

[B62] Yahagi S. Shimura K. Kasai T. (1996). An increase in cortical excitability with no change in spinal excitability during motor imagery. Percept. Mot. Skills 83, 288–290. 10.2466/pms.1996.83.1.288 8873203

[B63] Yamashita N. Moritani T. (1989). Anticipatory changes of soleus H-reflex amplitude during execution process for heel raise from standing position. Brain Res. 490, 148–151. 10.1016/0006-8993(89)90441-1 2758322

[B64] Zarkowski P. Shin C. J. Dang T. Russo J. Avery D. (2006). EEG and the variance of motor evoked potential amplitude. Clin. EEG Neurosci. 37, 247–251. 10.1177/155005940603700316 16929713

[B65] Zehr E. P. Stein R. B. (1999). Interaction of the Jendrassik maneuver with segmental presynaptic inhibition. Exp. Brain Res. 124, 474–480. 10.1007/s002210050643 10090659

